# Quantification of liver function using gadoxetic acid-enhanced MRI

**DOI:** 10.1007/s00261-020-02779-x

**Published:** 2020-10-09

**Authors:** Sarah Poetter-Lang, Nina Bastati, Alina Messner, Antonia Kristic, Alexander Herold, Jacqueline C. Hodge, Ahmed Ba-Ssalamah

**Affiliations:** Department of Biomedical Imaging and Image-Guided Therapy, General Hospital of Vienna (AKH), Medical University of Vienna, Waehringer Guertel 18-20, 1090 Vienna, Austria

**Keywords:** MRI, Gadoxetic acid, Diffuse liver diseases, Liver function, Quantification

## Abstract

The introduction of hepatobiliary contrast agents, most notably gadoxetic acid (GA), has expanded the role of MRI, allowing not only a morphologic but also a functional evaluation of the hepatobiliary system. The mechanism of uptake and excretion of gadoxetic acid via transporters, such as organic anion transporting polypeptides (OATP1,3), multidrug resistance-associated protein 2 (MRP2) and MRP3, has been elucidated in the literature. Furthermore, GA uptake can be estimated on either static images or on dynamic imaging, for example, the hepatic extraction fraction (HEF) and liver perfusion. GA-enhanced MRI has achieved an important role in evaluating morphology and function in chronic liver diseases (CLD), allowing to distinguish between the two subgroups of nonalcoholic fatty liver diseases (NAFLD), simple steatosis and nonalcoholic steatohepatitis (NASH), and help to stage fibrosis and cirrhosis, predict liver transplant graft survival, and preoperatively evaluate the risk of liver failure if major resection is planned. Finally, because of its noninvasive nature, GA-enhanced MRI can be used for long-term follow-up and post-treatment monitoring. This review article aims to describe the current role of GA-enhanced MRI in quantifying liver function in a variety of hepatobiliary disorders.

## Introduction

Imaging modalities such as ultrasound (US), computed tomography (CT), and conventional MRI are limited in their ability to detect chronic liver diseases (CLD) in their early stage, as structural changes often occur relatively late in the course of these diseases [1]. In recent years, the value of MRI in the diagnostic workup of hepatobiliary disorders has been significantly enhanced by three groups of contrast agents that increase the conspicuity of either focal or diffuse pathologic alterations and may be able to assess liver function [2].

(i) Conventional gadolinium chelates (nontissue-specific extracellular contrast agents) similar to iodinated contrast on CT, are typically given as a rapid intravenous bolus followed by dynamic imaging. Timing is critical to capture the arterial, portal venous, and equilibrium phases. Generalized liver enhancement reflects tissue perfusion, allowing the distinction between healthy and diseased liver, and thus helping to detect and characterize focal liver lesions. Likewise, conventional extracellular MR contrast agents are largely excreted via the kidneys [3, 4].
(ii) Gadolinium-based hepatobiliary contrast agents have dual uptake and excretion. Hepatocyte uptake, accounting for bile duct excretion, varies depending upon the specific hepatobiliary contrast agent, e.g., only 2–4% of gadobenate versus 50% of gadoxetic acid. The remaining injected dose behaves like extracellular contrast agents. Image acquisition after rapid bolus injection of gadobenate occurs much later (> 60 min rather than 20 min for gadoxetic acid), since slower hepatocyte uptake means increased time to accumulate adequate hepatobiliary contrast agent within the liver [2, 3, 5].
(iii) Superparamagnetic iron oxide (SPIO) contrast agents that are taken up by the Kupffer cells of the reticuloendothelial system [2].

In this article, we will focus on hepatobiliary contrast agents, and, in particular, gadoxetic acid (Gd-EOB-DTPA), also commercially available under the brand name Primovist® in the EU or Eovist® in the US. The multiparametric ability of gadoxetic acid-enhanced MR imaging provides morphologic and functional information about the hepatobiliary system, simultaneously. Multiparametric MRI is a comprehensive MRI that includes T1-, T2-weighted, and proton density fat fraction (PDFF) images, as well as magnetic resonance cholangiopancraticography (MRCP), diffusion-weighted imaging (DWI), and contrast-enhanced dynamic imaging, with a 20-min hepatobiliary phase and, eventually, additional MR elastography (MRE) depending on the radiologic center. The compilation of all these signal characteristics enables the determination of the composition and properties of focal or diffuse liver pathologies [6]. As a gadolinium-based paramagnetic MR contrast agent with dual elimination, approximately 50% of gadoxetic acid is excreted by the kidneys through glomerular filtration, while the other 50% is taken up by functional hepatocytes and excreted into the biliary system [7, 8]. After intravenous injection, GA is dispersed into the intra- and extravascular compartments during the arterial and portal venous phases, similar to conventional gadolinium chelates. But, subsequently, it is actively taken up by the hepatocytes during the transitional (3–5 min) and hepatobiliary phases (HBPs), 20 min after injection. Therefore, GA-enhanced MRI allows the synchronous evaluation of the hepatic vessels, biliary tree, and focal liver lesions [9–13] in addition to regional and total excretory liver function [14].

**Fig. 1:**
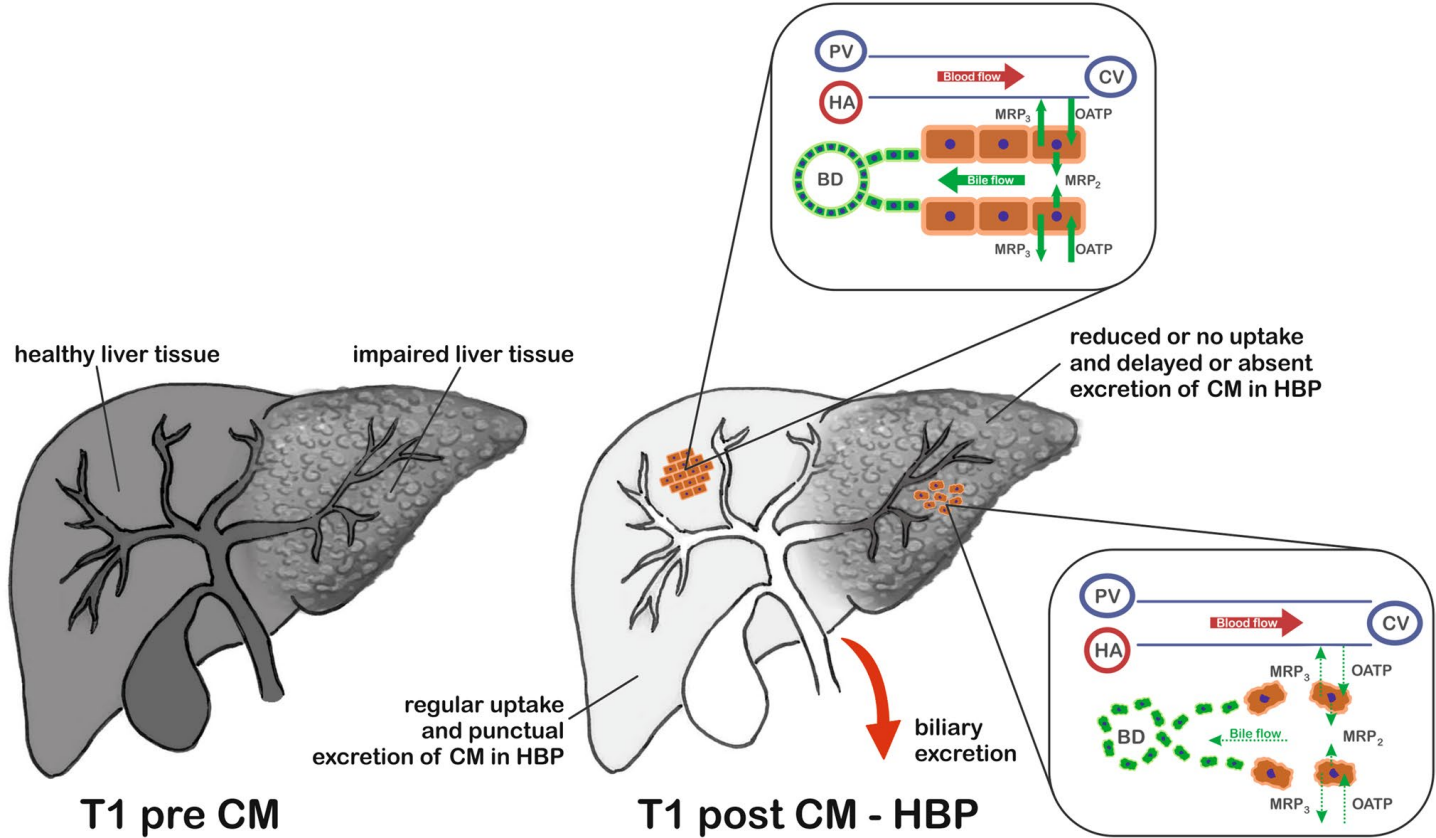
Liver divided into two halves: the first representing normally functioning parenchyma with normal uptake and timely excretion of the hepatobiliary contrast agent. The second half represents the cirrhotic, shrunken liver with irregular contours and architectural distortion due to fibrosis. Reduced or no uptake, and delayed or absent excretion of the hepatobiliary contrast agent indicates reduced liver function. Please also note the alteration in the hepatocyte membrane transporters, the so-called OATP, and MRP2 and 3

Evidence from basic research indicates that the uptake and excretion of GA is mediated by transmembrane transporters on hepatocytes [15, 16]. Gadoxetic acid enters the hepatocytes via active transport by organic anion transporting polypeptides (OATP1B1/3) and is excreted into the biliary tree via MRP2 [17]. Organic acid efflux from hepatocytes may also occur through the sinusoidal membrane because of bidirectional transport with OATP1B1/3 and MRP3 [17]. These insights into the cellular mechanism of transportation have led to a better understanding of how this unique contrast agent acts, as well as enabling the correlation of radiologic with histologic features of certain focal liver lesions, and also aiding in the staging of diffuse CLD [16, 18]. There are two main factors that determine the cellular uptake and excretion of gadoxetic acid (Fig. 1). First, alterations in OATPs (OATP1B1 and OATP1B3) expression, *affect* gadoxetic acid uptake [16]. Second, it also reflects the total number of hepatocytes. For instance, the decreased relative liver enhancement (RLE) in fibrosis and cirrhosis results from the fibrotic replacement of some normal hepatocytes of the liver parenchyma [19]. In particular, gadoxetic acid enhancement in the hepatobiliary phase (HBP) reflects the net difference between uptake and excretory transporter activity [20]. Based upon a variety of pharmacokinetic properties, including liver perfusion, vascular permeability, extracellular diffusion, and hepatocyte transporter expression, GA-enhanced MRI enables us to obtain a combination of morphologic and functional data simultaneously in normal and diseased hepatobiliary systems [21–25]. Therefore, gadoxetic acid is now considered a noninvasive biomarker of hepatobiliary disorders [6, 16].

In this article, we will describe the role of gadoxetic acid in the assessment of liver function in patients with diffuse CLDs using quantitative and semi-quantitative (i.e., qualitative features) scoring systems.

## Quantitative assessment of liver function

Visual analysis of the 20-min HBP GA-MR images provides the simplest estimate of liver function and has become an integral part of the radiology report [26–29]. More precise but tedious methods include (*E*_max_), time to peak (*T*_peak_), and elimination half-life (*t*_1/2_), which are semi-quantitative, and liver and vascular input concentration versus time curves, which are quantitative [8]. Additional further quantitative parameters include liver blood flow, blood volume, arterial and portal venous perfusion, and hepatocyte extraction fraction obtained by compartmental analysis or deconvolution [14, 24]. Despite their potential to become biomarkers of liver function, quantitative parameters remain *limited* by complex mathematical modeling, long examination times, and respiratory artifacts. Furthermore, with no standardization across MR scanners, no threshold values that could define normal and abnormal measures have been established. Therefore, these methods are still confined to research purposes.

**Fig. 2:**
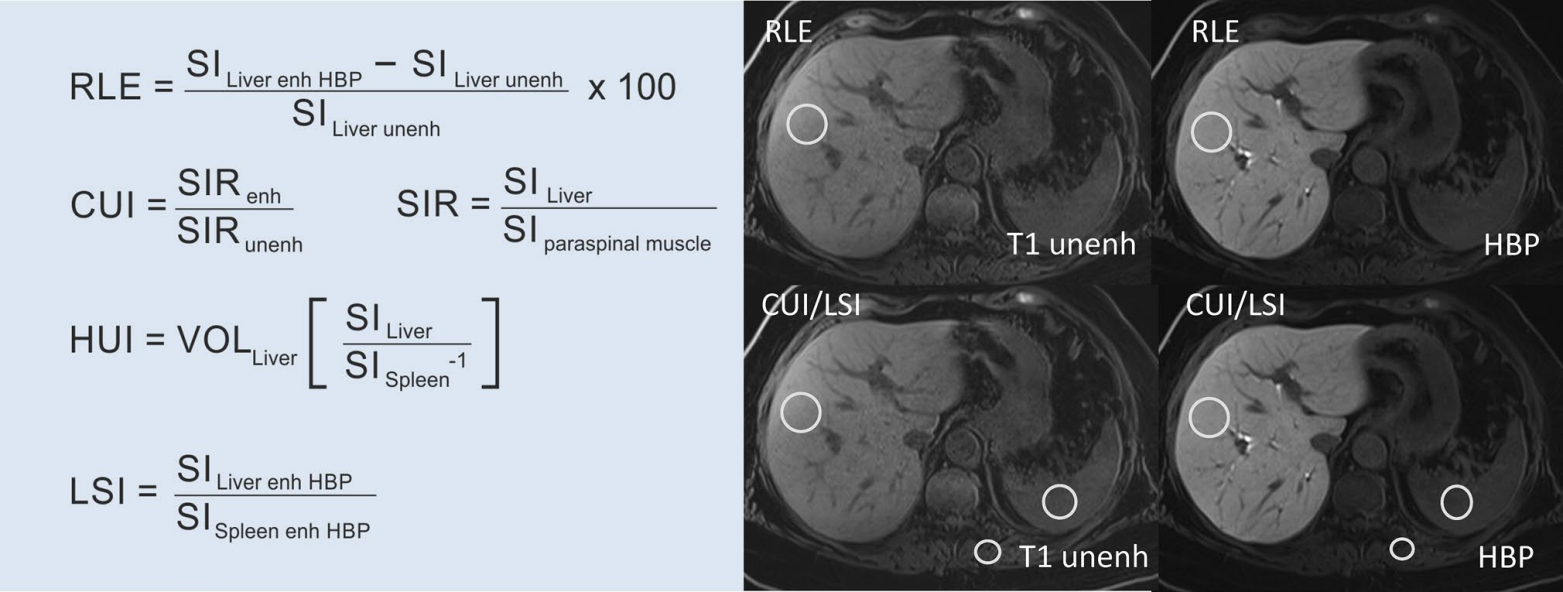
Equations for calculation of gadoxetic acid uptake for MRI scores. *RLE* relative liver enhancement, *CUI* contrast uptake index, *HUI* hepatic uptake index, *LSI* liver-spleen index, *SI* signal intensity, *SIR* signal intensity ratio, *enh* enhanced, *unenh* unenhanced

Several practical and simple quantitative imaging biomarkers of liver function have been introduced, obviating the need for the complex methods used in the animal models and human trials mentioned above. The relative liver enhancement (RLE), hepatic uptake index (HUI), contrast uptake index (CUI), and liver-to-spleen contrast index (LSI) [18, 29, 30] are such methods, relying on only two sequences: noncontrast, and 20-min HBP, which are both part of the routine MRI liver protocol. A simple equation yields any of these biomarkers, each of which has been shown to correlate with liver function parameters (Fig. 2). Most importantly, Beer et al. [18] found that all four of these scores showed a strong positive correlation with each other, answering the fierce debate as to which of these methods was superior [18]. Furthermore, our group confirmed that all four MRI-derived HPB scores correlated moderately with liver disease severity, as assessed by the Albumin-Bilirubin (ALBI) score, Model of End-Stage Liver Disease (MELD), and Child–Turcotte–Pugh (CTP) scores. These parameters were highly reproducible, with high inter-reader and intra-reader agreement. Two additional observations from this study should be mentioned. All four MRI parameters were fairly accurate at separating patients with significantly impaired liver function (MELD score ≥ 15) versus less-impaired (MELD < 15) liver function. This is of the outmost clinical importance, since a MELD [31] score of 15 is the threshold for liver transplantation, where the risk of dying from liver cirrhosis is greater than the post-transplantation mortality. Next, what stands out when considering these MR parameters in detail is the relatively high positive predictive value that each achieved, ranging from 0.876 for the RLE to 0.911 for the HUI. However, all these scores suffered from low negative predictive values, ranging between 0.447 and 0.532, which highlights their ability to validate rather than rule out liver dysfunction.

The new development of T1 mapping has more research than clinical applications, at present. There is a linear relationship between T1 relaxation times and contrast agent concentration. Therefore, T1 relaxation times should be more accurate than SI measurements derived from conventional T1 images [32, 33]. Haimerl et al. [34] have shown that GA-enhanced T1 relaxometry can be useful in diagnosing patients with impaired liver function or chronic liver disease, which they correlated with the Child–Pugh classification [35], the MELD score [31], and the ICG clearance test [36]. However, it should be noted that the relationship between T1 and GA concentration is somewhat more complex because GA is present in at least two compartments during the HBP, i.e., within hepatocytes and bile ducts, and may have different relaxivity rates within each compartment.

Although up to nine ROIs may be drawn, one for each segment, if 4a and 4b are considered as separate segments, four ROIs provide accurate values and are sufficient to reduce the heterogeneity seen in CLDs [24]. On a single level, ROIs of 2.0–5.0 cm^2^ encircle random, as-large-as-possible homogenous areas, avoiding large vessels, bile ducts, and focal lesions of both liver lobes, in addition to the spleen and nonatrophied left erector spinae. The mean value of these four liver ROIs is calculated.

Principally, it is still a subject of debate whether magnetic field strength (e.g., 1.5 or 3.0 Tesla (T)) influences quantitative MRI assessment, especially if contrast agents have been administered. Recently, Theilig et al. [37] reported that the HBP-derived RLE is constant over serial examinations, different scanner brands, as well as various field strengths. It should be noted that SI standardized to air, i.e., the signal-to-noise ratio, rather than absolute SI, is what matters. However, researchers should proceed with caution since 3 T MRI is known to give higher contrast-to-noise ratios than 1.5 T MRI for the liver parenchyma on contrast-enhanced MRI.

## Potential clinical uses for GA-enhanced MRI measures of liver function

### Nonalcoholic fatty liver disease (NAFLD)

Chronic liver diseases (CLD) are a major worldwide health problem. Although the prevalence of CLD from most etiologies has been stable or has even substantially declined, particularly in the case of hepatitis C virus (HCV) or hepatitis B virus (HBV) due to eradication or vaccination therapy, respectively [38], the prevalence of NAFLD has steadily increased, and is now the leading cause of CLD worldwide, affecting 80 to 100 million individuals in the USA alone [39, 40]. Therefore the early diagnosis of NAFLD and accurate assessment of liver disease severity are the keys to optimal patient management, where early treatment and lifestyle modification can arrest, and even reverse, deranged hepatic function [38] and histopathology [41].

**Fig. 3:**
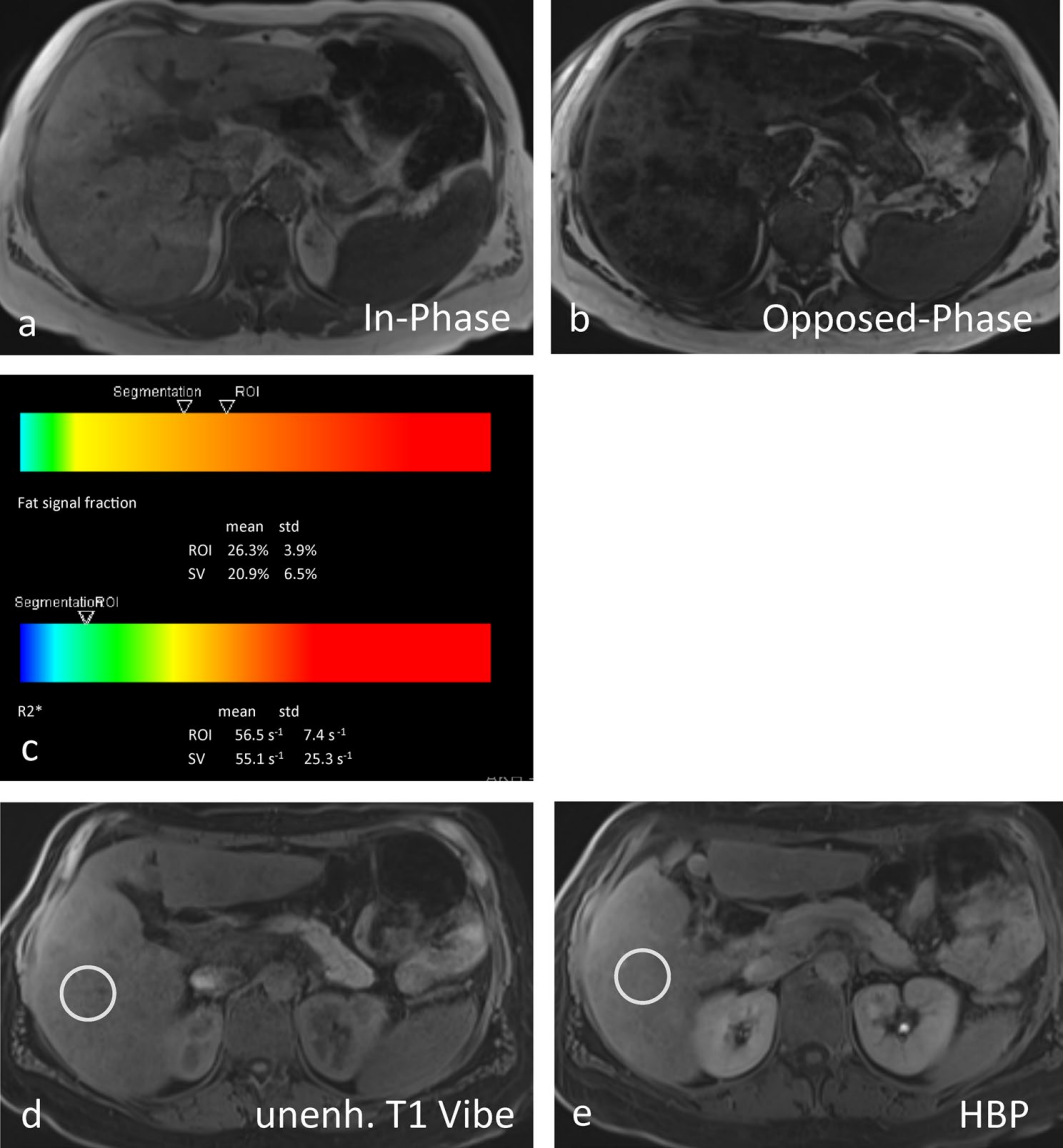
**a–e** A 37-year-old female patient with signs of massive inhomogeneous diffuse fatty infiltration of the liver with a significant signal drop from the in-phase image (**a**) compared to opposed phase image (**b**). The LiverLab (**c**) demonstrated moderate-to-severe steatosis with Inline statistics (mean, std) of PDFF and effective R2* over ROI and segmentation regions as text and a color bar. The mean value of the fat fraction was 26.3% and the effective R2* was 55.1 s^−1^, i.e., no significant iron overload. Consecutively, significantly reduced uptake and delayed excretion of Gadoxetic acid in the context of advanced impaired liver function. The measured RLE was 80% consistent with NASH and advanced fibrosis (**d**, **e**)

Bastati et al. [42] showed that the RLE, after GA administration, can differentiate simple steatosis patients from nonalcoholic steatohepatitis (NASH) patients. This method using cutoff value of 1.24 achieved an area under the receiver operator characteristic curve (AUROC) of 0.85 (95% CI 0.75–0.91). The authors suggested that this method may be a useful screening tool in selecting potential NAFLD patients for liver biopsy. However, despite its high sensitivity (97%), the specificity of RLE was only 63%, likely due to the liver fibrosis that was present in 68% of the cohort. As in many CLDs, fibrosis is a well-known confounder that decreases the RLE [43] **(**Fig. 3a–e). Similar, previous, retrospective studies [44, 45] and unpublished prospective data from our group confirmed the above-mentioned results.

### Liver fibrosis

**Fig. 4:**
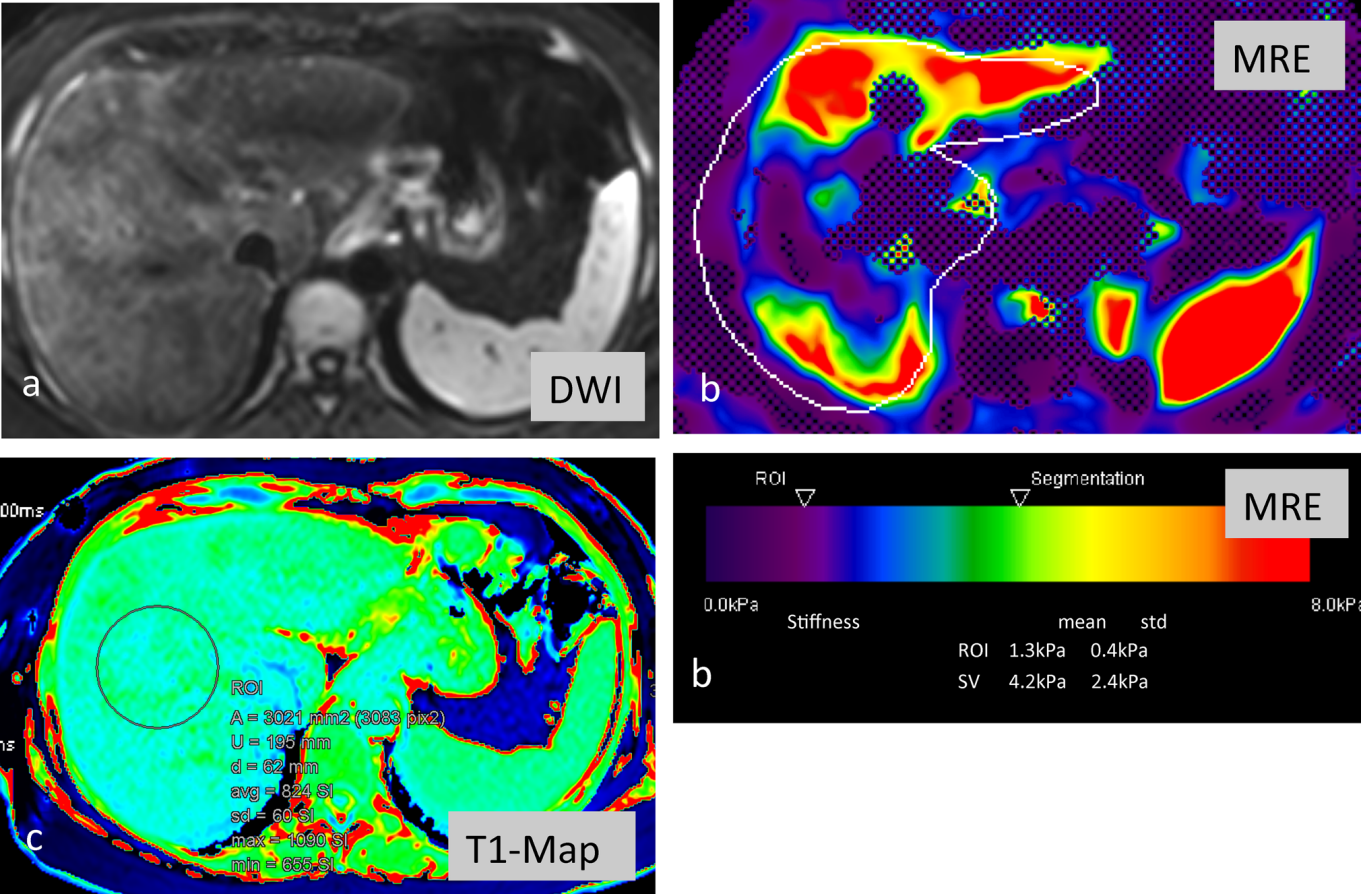
**a–c** Same patient from Fig. 3, with signs of NASH-associated advanced fibrosis/cirrhosis, with surface irregularities of the liver and increased signal intensity presumably due to edema and fibrosis in the diffusion-weighted images (**a**). The MR elastography (**b**) showed increased stiffness values of approximately 4 kPa, consistent with inflammation and fibrosis F4. Further new technical innovations, including T1 mapping (**c**), here, show increased values consistent with fibrosis

Liver fibrosis is a key determinant in the natural history of chronic liver diseases (CLDs) [46]. Per definition, liver fibrosis is a healing response to myriad injuries that result in CLD. Common insults include viral (A, B, C and D, E) and nonviral infections, alcohol, and NAFLD, while congenital hepatic fibrosis, Wilson’s disease, and a host of cholestatic liver diseases [47] are among the rare etiologies. Progressive liver fibrosis leads to end-stage liver cirrhosis as a result of fibrogenesis [48]. Moreover, because fibrosis may be reversible, early diagnosis is imperative so that early therapy can be instituted. Therefore, knowing the extent and degree of liver fibrosis in CLD patients is of clinical importance, as it can influence patient´s prognosis, surveillance, and treatment [48]. Liver biopsy currently remains the reference standard for the detection and staging of liver fibrosis, despite the risks associated with its invasiveness, inter-observer variability, potential for sampling errors, and poor patient acceptance, which makes it inappropriate for long-term monitoring or follow-up [49–51]. Routine biochemical and hematologic tests fail to help quantify liver fibrosis in approximately 50% of patients [51, 52]. There is a large body of literature describing the role of gadoxetic acid-enhanced MRI in the staging of liver fibrosis, using easily obtainable quantitative measures [53]. Feier et al. [54] found that RLE is a good discriminator for the presence of hepatic fibrosis, and Watanabe et al. have found that the contrast enhancement index (CEI) is an efficient biomarker for staging hepatic fibrosis [55]. Further studies have shown that RLE is an independent predictor of fibrosis and is highly accurate for staging F2 fibrosis and cirrhosis (stage F4), with AUROCs of 0.82 and 0.83, respectively [53]. Other groups have found that the hepatobiliary phase (HBP) T1 relaxation time significantly correlated with the fibrosis stage and necroinflammatory activity grade. However, although the HBP T1 relaxation time had a high diagnostic accuracy for stage 3 fibrosis (AUROC of 0.82), its diagnostic accuracy for grade 3 necroinflammatory activity was relatively low (AUROC of 0.68) [56, 57]. The authors found that HBP T1 relaxation times were better at differentiating stage 2 from stage 3 fibrosis than at distinguishing grade 2 from grade 3 necroinflammatory activity (Fig. 4a–c).

### Liver cirrhosis

Cirrhosis, the end-stage of hepatic fibrosis, is equivalent to stage 4 fibrosis in the Meta-analysis of Histological Data in Viral Hepatitis (METAVIR) system, or stages 5–6 in the Ishak scoring system [58], as well as in the newly introduced systems, including the Yale (Jain-Garcia), Laennec [59], and PIR systems [60].

Today, we know that cirrhosis is a dynamic process that may or may not progress. Indeed, early cirrhosis may be reversible. Thus, the former one-stage clinical and histologic description of “cirrhosis” is simplistic, and misrepresents current dogma [61]. Therefore, in the clinical setting, the term cirrhosis has been increasingly replaced by the term chronic liver disease (CLD), and thus, it is more important to distinguish between compensated CLD versus decompensated CLD, since, once patients cross this clinical boundary into decompensation, the odds are, by comparison, extremely low that they can avoid liver failure without transplantation or retransplantation [28, 62].

As a cure for chronic hepatitis C in responders where eradication of the viral load exceeds 95% post-treatment [63, 64], the focus of attention has now shifted to the regression of HCV-induced liver fibrosis, cirrhosis, and portal hypertension in these patients. Our group [65] hypothesized that the RLE might identify patients who have persistent hepatic inflammation and fibrosis, as well as portal hypertension despite HCV eradication. This is clinically relevant, as surveillance for portal hypertension and hepatocellular carcinoma are particularly important in patients with CLD**.** We evaluated changes in relative liver enhancement (RLE) obtained by GA-MRI in the hepatobiliary phase and changes in splenic volume (SV) after hepatitis C virus (HCV) eradication, as well as their predictive value for the development of (further) hepatic decompensation during follow-up. We observed an inverse correlation between the changes in RLE and SV (*P* < 0.001). In the non-responder patients, there was a decrease in RLE of − 11% (− 25% to − 3%; P = 0.019) and an increase in SV of 23% (7–43%; *P* = 0.004) (both *P* < 0.001 versus the responder patients). Interestingly, GA-MRI-nonresponse was associated with a substantially increased risk of (further) hepatic decompensation two years after the end of treatment: 80% versus 8%; *P* < 0.001. We concluded that GA-MRI might distinguish between individuals at low- and high-risk for (further) hepatic decompensation (GA-MRI-nonresponse) after HCV eradication. This could allow for individualized surveillance strategies.

The degree of parenchymal uptake and biliary excretion in GA-MRI may be useful for the evaluation of liver function as cirrhosis evolves [28, 66]. Tamada et al. found that hepatic parenchymal enhancement, i.e., RLE on GA-MRI, is affected by the severity of cirrhosis [67]. Tsuda and others, using RLE, also found that the liver’s signal intensity on the HBP of GA-MRI was lower in cirrhotic than in normal livers [68]. Conversely, Kanki et al. reported that the RLE derived from GA-MRI did not necessarily decrease with increased morphologic features of cirrhosis [69]. Other groups reported the value of T1 relaxometry in cirrhosis [70, 71], either unenhanced, or, after contrast media (CM) injection [72]. More recently, Sandrasegaran K. et al. [73] conducted a retrospective study to determine the value of quantitative parameters of GA-MRI in predicting prognosis in cirrhotics. Examining a cohort of 63 patients who had GA-MRI and a two-year clinical follow-up, they calculated the enhancement ratio (ER), contrast enhancement index (CEI), and contrast enhancement spleen index (CES). Comparing the usefulness of these parameters to clinical scores, such as the Child–Pugh score (CPS) and the model for end-stage liver disease (MELD) score, for predicting adverse outcomes, such as variceal bleeding (VB), hepatic encephalopathy (HE), and mortality at two years, they concluded that an ER of 15 or a CES of 20 were equivalent or better predictors of major morbidity and mortality than these commonly used clinical scores in cirrhotic patients.

### Preoperative evaluation of remnant liver function

Several studies have addressed the value of preoperative GA-MRI in predicting the risk of liver failure should major liver resection be performed [74–76]. This estimate of hepatic function reserve is crucial to avoid post-hepatectomy liver insufficiency (PHLI) [36, 74].

It is well known that, in the setting of metastatic liver disease, chemotherapy can adversely affect liver function. In particular, metastatic colorectal cancer (CRC) patients who receive specific drugs, such as oxaliplatin and or irinotecan, are predisposed to develop chemotherapy-associated steatohepatitis (CASH) and sinusoidal obstruction syndrome (SOS) [77, 78].

In those patients with less than 25–30% remnant liver function, PHLI is the major cause of postoperative morbidity and mortality. Because morphologic volume may overestimate functional volume [30, 74], volumetry alone (based on CT or conventional MRI) may not deliver an accurate prediction of PHLI in the presence of CLDs, where steatosis and/or fibrosis likely exist.

GA-MRI has proven superior for the estimation of both global and regional function, and studies have shown it to be a more reliable prognosticator than indocyanine green clearance (ICG) [30, 36].

In a retrospective study, our group [74] looked at how well functional future liver remnant (functFLR), as calculated from the RLE on GA-MRI and volumetry on multidetector computed tomography (MDCT) done within 10 weeks of a planned major resection, predicted post-hepatectomy liver failure (PHLF) following major liver resection compared to well-established clinical tests. In a multivariate analysis, we found that a decreased functFLR was independently associated with the probability of PHLF (0.561; *P* = 0.002). MRI software development is needed to eliminate the use of CT for volumetry, which is the major drawback of this technique.

Furthermore, Cho et al. showed that the RLE is an accurate discriminator for predicting which patients will develop PHLI after major liver resection [79]. Wibmer et al. found that decreased RLE in liver surgery candidates was associated with a higher risk of PHLI and perioperative mortality [30].

Kim DK et al. concluded that GA-MRI-derived measurements predicted PHLF better than the ICG clearance test in hepatocellular carcinoma (HCC) patients who underwent hepatectomy [36].

### Orthotopic liver transplantation (OLT)

Currently, GA-MRI and MR cholangiography have been increasingly used to assess liver graft dysfunction, since, unlike conventional gadolinium chelates, these techniques can provide both anatomic and functional information [80, 81]. Our group reported that, among the above-mentioned quantitative parameters derived from GA-MRI, the RLE is one of the most promising for graft evaluation [27]. Global and regional contrast media uptake and excretion of the graft can be calculated, providing the probability of graft survival. We could show that the RLE 20 min after contrast injection was directly related to the probability of one-year retransplantation-free survival in proportional hazard regression analysis (*P* = 0.005) [27].

## Qualitative or semi-quantitative assessment of liver function

### Qualitative assessment of OLT graft function

**Fig. 5:**
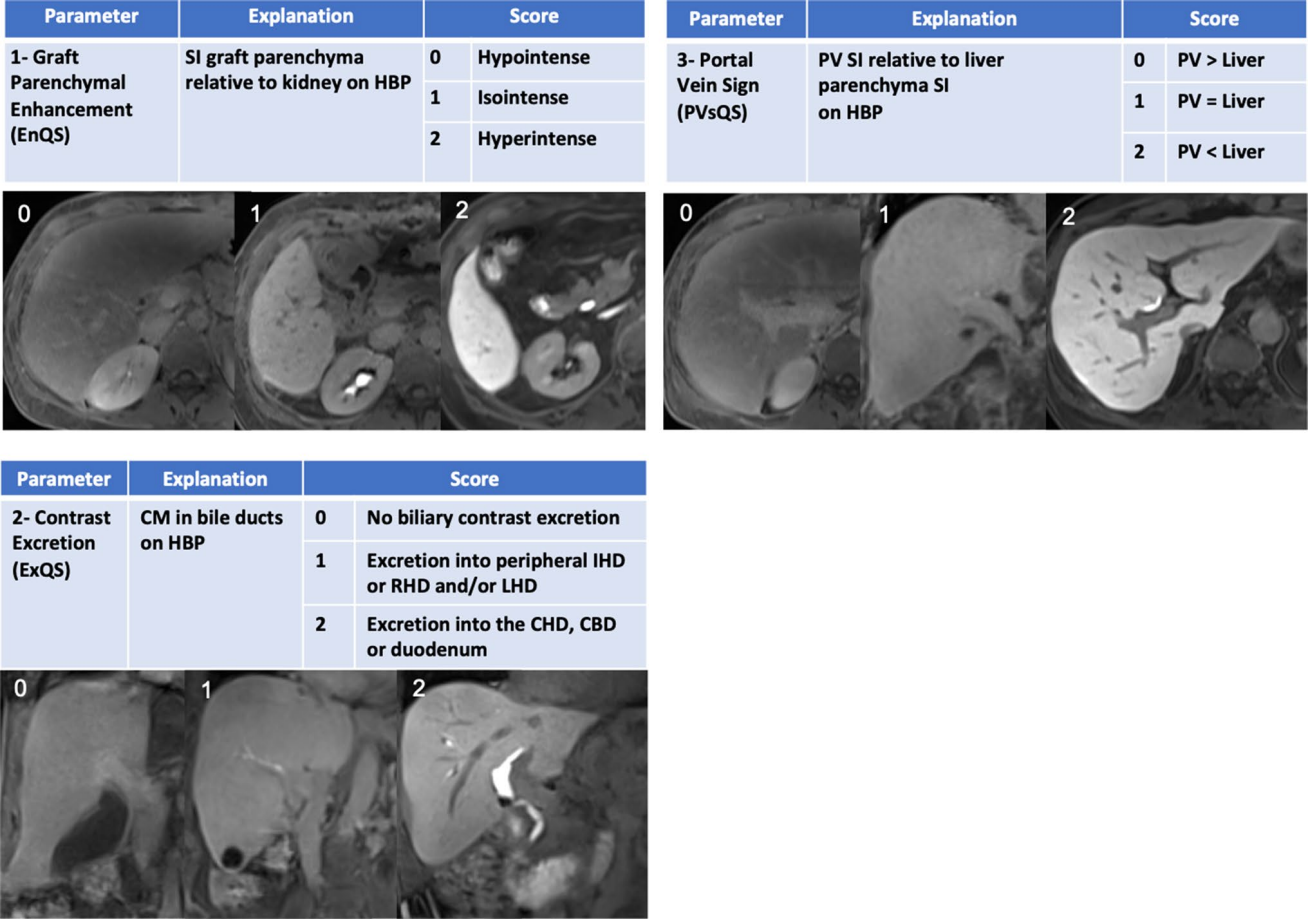
Functional liver imaging score (FLIS)-Score

Quantitative assessment of liver function using the calculation of the above-mentioned scores, such as RLE, is more tedious and time-consuming and normally cannot be performed as part of a routine MR examination. Furthermore, radiologists are used to assessing morphologic MR features and are less familiar with calculating lengthy and difficult equations. Therefore, Bastati et al. [27] introduced the functional liver imaging score (FLIS), derived from three hepatobiliary phase features on gadoxetic acid-enhanced MRI, each ranging from 0 to 2 on an ordinal scale. Following visual assessment of the liver parenchymal enhancement quality (EnQS), the rate of biliary contrast excretion (ExQS), and the persistence of contrast within the portal vein (PVsQS), the three scores are summed, and can range from 0 to 6, yielding the FLIS. Hence, the use of the term ‘semi-quantitative’ to describe the subjective and objective components that contribute to the FLIS (Fig. 5). Because the FLIS requires no signal intensity measurements, equations, or specific software, and is independent of MRI field strength and vendor, it can easily be incorporated into routine clinical practice.

### Qualitative assessment of liver function on CLD

Imaging techniques, including US, CT, and conventional MRI, have been used to diagnose cirrhosis and its sequelae based on morphological features. However, the prototypical features of cirrhosis bear no relationship to objective (i.e., functional) parameters of cirrhosis severity [6, 82]. Just as the FLIS, derived from GA-MRI, allows estimation of liver function in OLT grafts, it can be used in patients with chronic liver disease (CLD).

Previously described methods with which to assess the hepatobiliary phase uptake include the relative liver enhancement, the hepatic uptake index, the contrast enhancement index, and T1 relaxation values [18, 22, 24]. These methods all require complex computations and have vendor, field strength, and sequence dependencies that complicate their clinical application. FLIS is more practical for routine use than quantitative parameters, as it requires no SI measurements or calculations. The use of such an easy and reproducible score in clinical practice may potentially lead to better management of patients with CLD.

In a retrospective study, 265 patients with CLD, who had undergone GA-MRI, were assigned a FLIS score ranging from 0 to 6, based on the sum of three hepatobiliary phase features, as described above [28]. Patients were stratified into the following three groups according to fibrosis stage and a presence or history of hepatic decompensation: nonadvanced CLD; compensated advanced CLD (CACLD); and decompensated advanced CLD (DACLD). The predictive value of FLIS for first and/or further hepatic decompensation and for transplant-free survival, i.e., mortality, was investigated using Kaplan–Meier analysis, log-rank tests, and Cox regression analysis. Intra-observer and inter-observer intraclass correlation coefficients for FLIS were excellent, demonstrating the robustness and reproducibility of this scoring system [28].

**Fig. 6:**
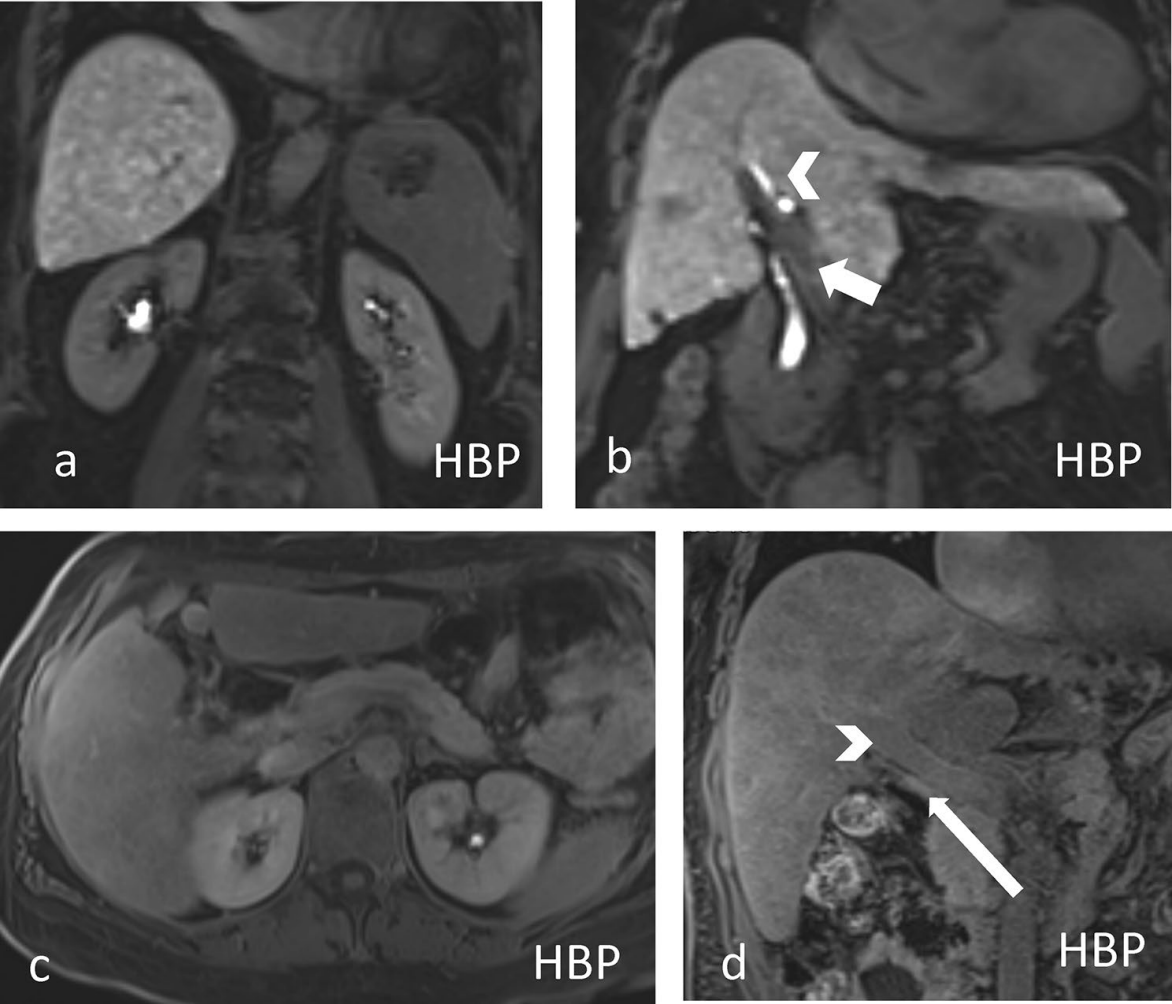
**a**, **b** Example of advanced chronic liver disease (ACLD) with preserved liver function. A 69-year-old female patient who developed HCC in NASH cirrhosis and underwent microwave ablation for HCC, with a 1 cm hypointense scar after therapy. The liver parenchymal enhancement was graded as 2 due to the good uptake, and the signal intensity of the liver, which was higher than that of the kidney on the HBP image (**a**). The contrast excretion was graded as 2 due to timely excretion based upon the presence of contrast media in the bile ducts, at 20 min after contrast injection (**b**, arrowhead). The portal vein sign was graded as 2, indicating an absence of signal intensity in the portal vein in the HBP (**b**, short arrow). In summary, the total sum of FLIS was a 6, which was indicative of preserved liver function. This patient with NASH-associated cirrhosis, is still alive, due to preserved liver function. **c**, **d** Example of ACLD with impaired liver function. Same patient from Fig. 3: The liver parenchymal enhancement was graded as 0 since the signal intensity of the liver parenchyma was less than that of the right kidney on HBP (**c**). The contrast excretion was graded as 0, as no contrast media was seen in the biliary tree 20 min after contrast injection (**d**, arrow). The portal vein sign was graded as 1 due to its almost isointense signal intensity to the liver (**d**, arrowhead). The total sum of the FLIS was 1, indicating very advanced, impaired liver function. 2 months later, the patient received liver transplantation

The results demonstrated that the FLIS is an independent predictor of liver-related events (e.g., first hepatic decompensation) and transplant-free survival (mortality) in patients with different causes of chronic liver disease (CLD). Both decompensated and compensated patients with CLD with a reduced FLIS showed a three- to seven-fold higher risk of mortality, respectively, even after adjusting for established prognostic factors (Fig. 6a–d).

GA has an excellent track record in focal liver lesion detection and characterization, as well as in liver function estimation. Transient severe motion (TSM) artifacts, which may degrade arterial-phase images in 18% of patients, on average [83], is the main limitation of GA. However, various techniques, including free-breathing, obtaining multiple arterial phases, saline dilution of GA, and/or slower injection rates, have successfully alleviated TSM [84, 85]. Furthermore, the 20-min delay between dynamic and HB phase imaging need not be wasted. By injecting GA at the start of the exam, dynamic imaging, T2 w-images, DWI, MR elastography, and other techniques, such as T2 mapping, can be performed while waiting. GA is preferable to gadobenate, not only because of the shorter interval between contrast injection and late-phase imaging but also because gadobenate’s negligible hepatocyte uptake limits its role in estimating liver function.

In conclusion, the estimation of liver function is a crucial clinical issue for identifying the broad spectrum of hepatobiliary disorders, for monitoring the progression of chronic liver disease (CLD), for determining the optimal therapeutic strategy, as well as for the prevention of post-interventional hepatic failure.

GA-MRI can be used to evaluate liver morphology, as well as calculate several quantitative parameters, including RLE, HUI, CEI, and the semi-quantitative FLIS parameter, which is independent of field strength, exam parameters, scanner type, or vendor.

## Abbreviations

ALBI: Albumin-Bilirubin score
AUROC: Area under the receiver operator characteristic curve
CACLD: Compensated advanced chronic liver diseases
CASH: Chemotherapy-associated steatohepatitis
CEI: Contrast enhancement index
CES: Contrast enhancement spleen index
CLD: Chronic liver diseases
CM: Contrast media
CPS: Child–Pugh score
CRC: Colorectal cancer
CT: Computed tomography
CTP: Child–Turcotte–Pugh score
CUI: Contrast uptake index
DACLD: Decompensated advanced chronic liver diseases
DWI: Diffusion-weighted imaging
ER: Enhancement ratio
FLIS: Functional liver imaging score
functFLR: Functional future liver remnant
GA: Gadoxetic acid
HBP: Hepatobiliary phase
HBV: Hepatitis B virus
HCC: Hepatocellular carcinoma
HCV: Hepatitis C virus
HE: Hepatic encephalopathy
HEF: Hepatic extraction fraction
HUI: Hepatic uptake index
ICG: Indocyanine green clearance
LSI: Liver-to-spleen contrast index
MDCT: Multidetector computed tomography
MELD: Model of End-Stage Liver Disease score
MRCP: Magnetic resonance cholangiopancraticography
MRE: MR elastography
MRI: Magnetic resonance imaging
MRP: Multidrug resistance-associated protein
NAFLD: Nonalcoholic fatty liver diseases
NASH: Nonalcoholic steatohepatitis
OATP: Organic anion transporting polypeptides
PDFF: Proton density fat fraction
PHLF: Post-hepatectomy liver failure
PHLI: Post-hepatectomy liver insufficiency
RLE: Relative liver enhancement
SOS: Sinusoidal obstruction syndrome
SPIO: Superparamagnetic iron oxide
SV: Splenic volume
TSM: Transient severe motion
US: Ultrasound
VB: Variceal bleeding
